# Clinical Asthma Remission Obtained with Biologics in Real Life: Patients’ Prevalence and Characteristics

**DOI:** 10.3390/jpm13061020

**Published:** 2023-06-20

**Authors:** Bruno Sposato, Francesco Bianchi, Alberto Ricci, Marco Scalese

**Affiliations:** 1 Pneumology Department, Azienda USL Toscana Sud-Est, “Misericordia” Hospital, Via Senese 161, 58100 Grosseto, Italy; 2 Division of Pneumology, Department of Clinical and Molecular Medicine, Sapienza University of Rome, AOU Sant’Andrea, 00189 Rome, Italy; 3 Clinic Physiology Institute, National Research Centre, 56124 Pisa, Italy

**Keywords:** severe asthma, biologic, remission, real life

## Abstract

Background: The prevalence of clinical asthma remission with biologics in severe asthma has not been well understood yet. We do not even know whether there might be characteristics that identify subjects prone to remission of the disease. Materials and Methods: Retrospectively, four groups of severe asthmatics already treated with Omalizumab, Mepolizumab, Benralizumab and Dupilumab (302, 55, 95 and 34 patients, respectively) for at least 12 months were considered. The number of individuals with clinical asthma remission was sought in each group. This was considered when patients, after a treatment of at least 1 year with one of the aforesaid biologics, showed the disappearance of asthma symptoms (ACT ≥ 20), zero exacerbations, suspension of oral corticosteroids and a FEV_1_% ≥ 80%. Baseline characteristics of patients with and without remission were also taken into account. Results: The prevalence of asthma remission after a mean of 37.8 ± 19.2, 13.5 ± 1.7, 15.4 ± 5.5 and 12 ± 0 months of Omalizumab, Mepolizumab, Benralizumab and Dupilumab treatments was 21.8%, 23.6%, 35.8% and 23.5%, respectively. For each biologic, different baseline characteristics, seem to be associated with failure to achieve clinical asthma remission. Older age, higher BMI, a later age of asthma onset, rhinitis/sinusitis/nasal polyposis, comorbidities and a greater asthma severity may be the characteristics of a suboptimal response to biologic treatments. Conclusion: All biologics have the potential to induce disease remission in severe asthmatics. For each biologic, there may be several markers that can identify the patients who will not achieve asthma remission. It would be important to detect them (by carrying out targeted studies) as they would allow us to select the best biologic that may induce clinical asthma remission on a larger number of patients.

## 1. Introduction

Asthma may progress over time, but it may also undergo remission. Clinical asthma remission is possible as part of asthma natural history and its prevalence in adult asthmatics may vary between 2% and 52% [1]. It is characterised by a high level of disease control (absence of symptoms and exacerbations), no use of oral corticosteroids and normalisation or optimisation of lung function with or without ongoing treatment [1].

The factors associated with remission include mild asthma, better lung function, a higher level of asthma control, younger age, early-onset asthma, shorter disease duration, milder bronchial hyperresponsiveness, fewer comorbidities and smoking cessation or never smoking [1].

With the advent of biologic therapies, directed toward the regulation of airway inflammation, asthma control has greatly improved, especially in the more severe forms of the disease. In fact, biologics are highly effective in reducing exacerbations, diminishing symptoms and improving lung function in well-defined asthma populations. Therefore, it is possible to achieve asthma remission with biologics. As already said, according to a recent definition, clinical asthma remission should be characterised by the absence of symptoms and exacerbations, no use of oral corticosteroids and normalisation of lung function (FEV_1_ ≥ 80%) after at least one year during treatment with biologic. When there is also a normalisation of the underlying pathology (e.g., resolution of airway inflammation), we are faced with a complete remission [1]. It is not clear what the prevalence might be and what characteristics might predict “clinical asthma remission” in patients with a severe form of the disease treated with biologics in real life. The few studies addressing this topic highlight that the prevalence of “super responders” (patients who are exacerbation-free and off OCS at one year) to all anti-IL5 (Mepolizumab, Benralizumab, Reslizumab) after two years of treatment, was 14% [2], whereas these prevalence values were 28.3% [3] and 39–46.3% [4,5] when treated with Mepolizumab and Benralizumab, respectively, for approximately 12 months. In the only two studies dealing with this topic, clinical asthma remission (according to the aforementioned criteria) was observed in 15 [6] and 21.7% [7] of cases treated with Benralizumab for one and two years, respectively. Recently, a study conducted in real life has shown that the prevalence of clinical asthma remission obtained with Mepolizumab and Benralizumab was 30.12% and 40%, respectively [8]. There does not seem to be any studies on asthma disappearance obtained with other biologics in the literature. Low ACQ, nasal polyp, adult onset asthma and FEV1 > 80% were predictive factors for asthma remission after treatment with Benralizumab or Mepolizumab [2,3,4,5,6,7,8,9].

Given the limited knowledge on this topic, we wanted to investigate the prevalence of clinical asthma remission and the baseline characteristics that could predict it in subjects with severe asthma treated with Omalizumab, Mepolizumab, Benralizumab and Dupilumab.

## 2. Materials and Methods

In this study, we retrospectively performed a post hoc analysis of four groups of patients already treated with Omalizumab, Mepolizumab, Benralizumab and Dupilumab. All patients recruited in this study had to be on therapy with these biologics for at least 12 months and all they had to be also responsive to treatments. They had already been studied in other research with the aim of verifying the effectiveness of the above-mentioned biologics in real life. All subjects had a severe asthma diagnosis, fulfilling all the diagnostic criteria established by the guidelines [10]. Their asthma had been poorly controlled even while using high ICS doses, long-acting bronchodilators, anti-leukotrienes (montelukast) and/or OCs, which made it necessary to add a biologic, as recommended by step 5 of the GINA asthma guidelines. All patients had to be adherent to the inhaled treatments and had to use devices correctly. Omalizumab was prescribed to patients that had an allergic sensitization to perennial allergens and a baseline serum IgE > 76 UI/mL. Benralizumab or Mepolizumab was prescribed to subjects that showed a peripheral blood eosinophil count above 300/μL and more than 150/μL in patients continuously or frequently treated with oral corticosteroids (OCS) before the biologic treatment. A blood eosinophil count ≥150 cells/μL or FeNO ≥ 25 ppb associated to lifelong or near-continuous OCS therapies was required for the prescription of Dupilumab.

A positive response to biologics was considered if such therapy continued for at least 12 months. For each group, the number of patients with clinical asthma remission was sought at the end of each individual’s treatment period, by using the strictest criteria [1]. This was considered when patients, after a treatment of at least 1 year with one of the aforesaid biologics, showed the disappearance of asthma symptoms (ACT ≥ 20), zero exacerbations, suspension of OCS and a FEV_1_% ≥ 80%. Subsequently, we evaluated the anthropometric and clinical/biologic characteristics observed before the biologic treatments which were compared in subjects with and without “clinical asthma remission” in order to identify possible characteristics/markers that could allow us to classify subjects who could obtain disease remission by using biologics.

Age, gender, BMI, smoking habit, age of asthma onset, allergies, rhinitis/sinusitis/polyposis presence and other comorbidities were taken into account. We also considered the Asthma Control Test (ACT), FEV_1_%, FEF_25–75_%, blood eosinophils, serum IgE, FENO, OCS use and a possible previous treatment with another biologic in subjects with asthma remission and in those who never showed a complete disappearance of this disease. This article is based on data of previously conducted research studies (post-hoc analysis) whose protocols had been approved by the various ethical committees.

### Statistical Analysis

Categorical variables are stated as number (n) and percentage (%). Normally distributed continuous variables are expressed as mean ± standard deviation. Fisher exact or Chi-Square tests were used for comparisons of categorical variables. Mann Whitney tests were used to compare continuous variables. A *p*-value < 0.05 was considered statistically significant.

## 3. Results

**Figure 1:**
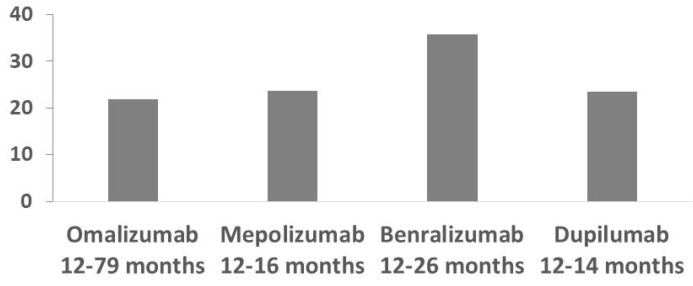
Asthma remission prevalence obtained at different times for Omalizumab, Mepolizumab, Benralizumab and Dupilumab treatments.

We considered 302, 55, 95 and 34 patients that were treated with Omalizumab, Mepolizumab, Benralizumab and Dupilumab. The prevalence of clinical asthma remission after a mean of 37.8 ± 19.2, 13.5 ± 1.7, 15.4 ± 5.5 and 12 ± 0 months of Omalizumab, Mepolizumab, Benralizumab and Dupilumab treatments was 21.8%, 23.6%, 35.8% and 23.5%, respectively (Figure 1). In patients treated with Omalizumab, older age, higher BMI, a later age of asthma onset, sinusitis/nasal polyposis, hypertension and chronic heart disease presence and higher exacerbation numbers may predict failure to achieve asthma remission (Table 1). There was no difference between the number of subjects who achieved this goal after 12–24, 25–48 and >49 months of Omalizumab treatment (21 [31.8%], 24 [36.4%] and 21 [31.8%] subjects, respectively; *p* = 0.87). Patients with more severe asthma (lower FEV_1_%, FEF_25–75_% and consequently more exacerbations) may have a reduced possibility to fully recover with Mepolizumab. On the contrary, subjects with higher FENO are those who could achieve remission when treated with Mepolizumab. Higher BMI and rhinitis may be the characteristics of a suboptimal response to Benralizumab. The analysis of the characteristics of patients treated with Dupilumab does not highlight any difference between individuals who showed asthma remission and those who had a suboptimal response with this biologic.

## 4. Discussion

This study basically highlighted that clinical asthma remission can be obtained with all biologics in a significant number of subjects. Therefore, it is possible to obtain a complete clinical disappearance of asthma even in patients who were uncontrolled before using biologics (severe asthma phenotype). In fact, these subjects showed normalisation of lung function, absence of exacerbations/symptoms and suspension of OCS during the treatment period considered in our study with the various biologics. This overturns the concept of “irreversibility” of inflammation and bronchial obstruction in “severe asthma phenotype” existing before the advent of biologics in the treatment of this disease. Therefore, they can modify the natural history of asthma up to a complete remission of the disease. What we should improve on is the precision in prescribing these drugs, which could be done by looking for markers that are more refined. In fact, the various asthma phenotypes may overlap in many patients; therefore, it may be often difficult to choose the right biologic, which may lead to possible repercussions on a reduced treatment efficacy.

To date, there are few data on the prevalence of asthma remission in real life with individuals treated with monoclonal antibodies. Recently, a real-life study has shown that the prevalence of clinical asthma remission obtained with Mepolizumab and Benralizumab was similar to that observed in our research [8]. It is interesting to note that, according to our and other studies [6,8], after just 1 year of treatment, a significant number of subjects may undergo remission of the disease with biologics. This makes us reflect on the rapidity of action of these drugs in inducing asthma remission in several subjects. We should try to understand whether the number of subjects who achieved it could be made progressively higher over time.

Although a higher prevalence of clinical asthma remission seemed to be obtained with Benralizumab, the results actually observed with the various treatments are not comparable with one another as the characteristics of the patients at baseline, and especially the times of therapy, are different among the various groups. Although all subjects had been on treatment for at least 1 year, the duration of Mepolizumab and Dupilumab therapy was shorter than that of Benralizumab. The prevalence of disease remission might have been underestimated with the first two above-mentioned treatments.

However it should also be noted that an assessment of asthma remission after a period of only 12 months of follow up could be limiting. Probably longer periods could confirm with greater certainty the achievement of the complete disappearance of asthma. This may be a limitation of the study as it may have influenced the results.

Clinical asthma remission was observed in approximately 21% of Omalizumab-treated asthmatics after approximately a median of 3 years of therapy. A prolonged therapy does not seem to change the prevalence of patients with asthma remission as, apparently, the such biologic is effective from the first months of treatment, remaining stable in time. Several patients receiving Omalizumab had baseline eosinophil values > 300 cells/µL. Probably, a selection of predominantly allergic individuals, rather than subjects with overlapping allergic/eosinophilic asthma, could lead to a higher prevalence of remission. Omalizumab is able to reduce the number of eosinophils but not as much as the other biologics currently available in Italy [11]. An inadequate eosinophil reduction may lead to a poor response to treatment [12].

GINA guidelines [10] indicate which type of biologic has to be chosen (to obtain an optimal clinical response) on the basis of the asthma phenotype (eosinophilic or allergic) and according to characteristics such as the age of asthma onset, the presence of polyposis, the number of exacerbations, the use of systemic steroids, etc. Our study also highlights some features for each biologic that could suggest the type of monoclonal antibody to be used in order to obtain clinical asthma remission.

We have observed that younger patients with youth-onset asthma, low BMI, few comorbidities and fewer exacerbations are likely to experience disease remission when treated with Omalizumab. After only about 1 year of treatment, a quarter of the patients treated with Mepolizumab can show asthma remission. It is possible that with the prolongation of treatment the prevalence would be even higher. In fact, some authors have observed a progressive and lasting improvement of asthma in real life as well as an increase of the number of super-responders prolonging treatment with Mepolizumab over time [3,13]. Patients with more severe asthma, i.e., more impaired lung function and consequently more exacerbations, may be less likely to obtain a complete disappearance of asthma with Mepolizumab. This could be observed with all biologics; it is obvious that less severe patients may have a better response than more severe ones and this could happen with all biologics [1]. Higher FENO levels may be predictive of achieving asthma remission when we use Mepolizumab. This agrees with another real-life study where adults with severe eosinophilic asthma and who had a baseline FeNO ≥ 50 ppb experienced a greater decrease in exacerbations after 12 months of anti-IL-5 or IL-5R biologic than those with a FeNO < 50 ppb [14].

In our study, we observed a higher remission prevalence with Benralizumab in contrast to what was found by some authors [6,7] but comparable with what observed by others [8]. The clearing of eosinophils, which Benralizumab can perform, may play a role in achieving asthma remission more effectively. This may more frequently be observed in subjects with rhinitis and low BMI, which may be traits that could identify subjects more prone to a clinical complete disappearance of asthma.

Dupilumab also appears to be efficacious in about 25% of cases in inducing asthma disappearance. An evaluation time limited to only 12 months and a low number of cases could have underestimated the results. There are no other studies in the literature about clinical asthma remission with Dupilumab. From our analysis, there are apparently no markers that can predict such a remission. However, possible predictive remission markers may not be highlighted because of the limited number of the patients considered. Therefore, this aspect should be studied in a larger number of subjects treated with Dupilumab.

We think that identifying markers of asthma remission, rather than response on some outcomes, could allow us to perform a more refined phenotyping of patients so as to be able to better choose the right type of biologic to use. This could lead us to obtain asthma remission in a larger number of individuals.

## 5. Conclusions

All biologics have the potential to induce disease remission in severe asthmatics. Young age, youthful disease onset, low BMI, few comorbidities and few exacerbations observed at the beginning of therapy may be features associated with clinical asthma remission when Omalizumab is used. Patients that showed a more impaired lung function at baseline may not be prone to complete clinical asthma disappearance with Mepolizumab. On the contrary, higher baseline FENO levels may be predictive of achieving clinical asthma remission when we use Mepolizumab. This may also be observed in subjects with rhinitis and low BMI when treated with Benralizumab. Apparently, there are no markers that could predict clinical asthma remission with Dupilumab. Therefore, for each biologic, there may be several markers that can predict it. Targeted studies aimed at identifying these indicators more precisely are needed, as they would allow us to select the best biologic that may lead to us obtaining not only a better clinical response to treatment but also a “complete clinical asthma remission” on a larger number of patients.

## Figures and Tables

**Table 1:** Baseline characteristics of patients (prior to biologic treatments) with or without asthma remission after at least one year of Omalizumab, Mepolizumab, Benralizumab and Dupilumab therapy.

	OMALIZUMAB			MEPOLIZUMAB			BENRALIZUMAB			DUPILUMAB		
	AR (66–21.8%)	Non AR (236–78.2%)	*p*	AR (13–23.6%)	Non AR (42–76.4%)	*p*	AR (34–35.8%)	Non AR (61–64.2%)	*p*	AR (8–23.5%)	Non AR (26–76.5%)	*p*
Age	**48.4 ± 11.7**	**53.9 ± 13.4**	**0.001**	57.1 ± 13.5	56.9 ± 13.7	0.996	55.9 ± 11.2	59.2 ± 12.6	0.165	58.3 ± 12.2	59.8 ± 12.7	0.439
Sex (M/F)	27/39	77/159	0.210	6/7	17/25	0.716	13/21	23/38	0.959	2/6	12/14	0.514
Treatment time	37.8 ± 19.2	37.9 ± 19.4	0.992	13.5 ± 1.7	14.4 ± 2.6	0.322	**15.4 ± 5.5**	**20.4 ± 7.3**	**0.010**	**12 ± 0**	**13 ± 0.96**	**0.017**
Smokers	19 (28.8%)	51 (21.6%)	0.221	0 (0%)	3 (7.1%)	0.770	2 (5.9%)	3 (4.9%)	0.781	0 (0%)	0 (0%)	---
BMI	**25.9 ± 4.8**	**27.1 ± 4.7**	**0.054**	24.6 ± 2.38	26.1 ± 4.1	0.239	**25.5 ± 4.1**	**27 ± 4.1**	**0.059**	25.5 ± 2.1	27.5 ± 3.7	0.486
Obese subjects	10 (15.1%)	56 (23.7%)	0.136	0 (0%)	8 (19.0%)	0.210	5 (14.7%)	13 (21.3%)	0.430	0 (0%)	4 (15.4%)	0.579
Age of asthma onset	**24.8 ± 15.8**	**29.9 ± 17.4**	**0.026**	43.7 ± 14.6	36.1 ± 16.5	0.109	35.1 ± 14.4	36.6 ± 14.6	0.623	32.7 ± 15.7	35.5 ± 19.6	0.810
House dust mite	54 (81.8%)	205 (86.9%)	0.299	5 (38.4%)	15 (35.7%)	0.857	11 (32.3%)	18 (29.5%)	0.773	1 (12.5%)	13 (50%)	0.140
Pollens	43 (65.1%)	148 (62.7%)	0.716	1 (7.7%)	16 (38.1%)	0.083	11 (32.3%)	21 (34.4%)	0.837	2 (25%)	14 (53.8%)	0.305
Cat/dog dander	22 (33.3%)	70 (29.7%)	0.566	1 (7.7%)	5 (11.9%)	0.933	5 (14.7%)	6 (9.8%)	0.477	0 (0%)	4 (15.4%)	0.579
Moulds	9 (13.6%)	43 (18.2%)	0.383	**4 (30.7%)**	**1 (2.4%)**	**0.010**	1 (2.9%)	5 (8.2%)	0.569	0 (0%)	3 (11.5%)	0.769
Rhinitis	42 (63.6%)	161 (68.2%)	0.483	8 (61.5%)	24 (57.1%)	0.778	**21 (61.8%)**	**25 (41%)**	**0.052**	5 (62.5%)	20 (76.9%)	0.726
Sinusitis/polyposis	**17 (25.7%)**	**116 (49.2%)**	**0.001**	8 (61.5%)	29 (69)	0.614	22 (64.7%)	30 (49.2%)	0.145	5 (62.5%)	18 (69.2%	0.939
Hypertension	**14 (21.2%)**	**90 (38.1%)**	**0.010**	2 (15.4%)	13 (31%)	0.456	4 (11.8%)	16 (26.2%)	0.163	0 (0%)	5 (19.2%)	0.439
Chronic heart disease	**1 (1.5%)**	**37 (15.7%)**	**0.004**	0 (0%)	2 (4.7%)	0.963	2 (5.9%)	4 (6.6%)	0.756	0 (0%)	1 (3.8%)	0.526
Diabetes	3 (4.5%)	17 (7.2%)	0.625	1 (7.7%)	3 (7.1%)	0.586	1 (2.9%)	3 (4.9%)	0.942	0 (0%)	0 (0%)	---
Osteoporosis	5 (7.6%)	26 (11%)	0.415	1 (7.7%)	9 (21.4%)	0.477	1 (2.9%)	7 (11.5%)	0.293	2 (25%)	4 (15.4%)	0.925
Gastro-esophageal reflux	19 (28.8%)	90 (38.1%)	0.162	3 (23.1%)	16 (38.1%)	0.508	10 (29.4%)	23 (37.7%)	0.415	2 (25%)	5 (19.2%)	0.883
OSAS	3 (4.5%)	7 (3%)	0.806	0 (0%)	1 (2.4%)	0.531	0	5 (8.2%)	0.216	0 (0%)	0 (0%)	---
Mental disorders	3 (4.5%)	27 (11.4%)	0.154	4 (30.7%)	14 (33.3%)	0.868	8 (23.5%)	21 (34.4%)	0.268	0 (0%)	3 (11.5%)	0.769
Exacerbations	**2.62 ± 1.25**	**3.13 ± 1.41**	**0.007**	**3.31 ± 1**	**4.5 ± 2.1**	**0.037**	4.6 ± 2.7	3.8 ± 2.1	0.156	4 ± 0.9	4.5 ± 2.4	0.890
ACT	15.9 ± 4.7	14.9 ± 5.1	0.439	15.7 ± 4.6	13.3 ± 3.7	0.052	15.2 ± 4.3	15.7 ± 4.3	0.464	14.6 ± 1.4	14.7 ± 2.2	0.897
FEV_1_ %	72.1 ± 15.6	69.5 ± 19.9	0.121	**83.4 ± 10.1**	**67.7 ± 19.1**	**0.005**	73.7 ± 22.5	75.1 ± 26.8	0.854	76.1 ± 14.5	65.2 ± 20.3	0.183
FEF_25–75_%	42.3 ± 23.6	40.1 ± 19.8	0.786	**46.4 ± 13.7**	**28.7 ± 20**	**0.006**	44.2 ± 20.8	50 ± 27.8	0.478	33.3 ± 8.7	34.7 ± 12.9	0.506
Total serum IgE UI/ml	374.8 ± 292.2	385.6 ± 319.2	0.797	275 ± 298.5	373.2 ± 647.4	0.971	440.3 ± 729.6	459.3 ± 739.0	0.829	119.7 ± 72	364 ± 481	0.081
Blood eosinophils (n/µL)	314.5 ± 271	357.5 ± 288.7	0.457	574 ± 329	839.8 ± 1054	0.407	993.5 ± 728	808.6 ± 689.4	0.112	500 ± 158.4	607 ± 328.9	0.661
FENO (ppb)	36.4 ± 32.3	35 ± 40.2	0.752	**76.2 ± 50.7**	**41.3 ± 22.3**	**0.050**	51.5 ± 31.5	45.9 ± 36.4	0.376	53.7 ± 5.1	54.1 ± 15.6	0.904
Continuous oral corticosteroids use (pre-treatment)	7 (10.6%)	33 (18.2%)	0.474	10 (76.9%)	35 (83.3%)	0.911	19 (55.6%)	37 (60.7%)	0.650	4 (50%)	17 (65.4%)	0.713
Subjects with a previous treatment with other biologics	-	-		2 (15.4%)	8 (19%)	0.910	5 (14.7%)	14 (23%)	0.335	4 (50%)	12 (46.1%)	0.830

## Data Availability

All data generated or analysed during this study are included in this article. Further inquiries can be directed to the corresponding author.
